# Opposite Effects of Early Maternal Deprivation on Neurogenesis in Male versus Female Rats

**DOI:** 10.1371/journal.pone.0003675

**Published:** 2009-01-30

**Authors:** Charlotte A. Oomen, Carlos E. N. Girardi, Rudy Cahyadi, Eva C. Verbeek, Harm Krugers, Marian Joëls, Paul J. Lucassen

**Affiliations:** SILS Centre for Neuroscience, University of Amsterdam, Amsterdam, The Netherlands; James Cook University, Australia

## Abstract

**Background:**

Major depression is more prevalent in women than in men. The underlying neurobiological mechanisms are not well understood, but recent data shows that hippocampal volume reductions in depressed women occur only when depression is preceded by an early life stressor. This underlines the potential importance of early life stress, at least in women, for the vulnerability to develop depression. Perinatal stress exposure in rodents affects critical periods of brain development that persistently alter structural, emotional and neuroendocrine parameters in adult offspring. Moreover, stress inhibits adult hippocampal neurogenesis, a form of structural plasticity that has been implicated a.o. in antidepressant action and is highly abundant early postnatally. We here tested the hypothesis that early life stress differentially affects hippocampal structural plasticity in female versus male offspring.

**Principal Findings:**

We show that 24 h of maternal deprivation (MD) at PND3 affects hippocampal structural plasticity at PND21 in a sex-dependent manner. Neurogenesis was significantly increased in male but decreased in female offspring after MD. Since no other structural changes were found in granule cell layer volume, newborn cell survival or proliferation rate, astrocyte number or gliogenesis, this indicates that MD elicits specific changes in subsets of differentiating cells and differentially affects immature neurons. The MD induced sex-specific effects on neurogenesis cannot be explained by differences in maternal care.

**Conclusions:**

Our data shows that early environment has a critical influence on establishing sex differences in neural plasticity and supports the concept that the setpoint for neurogenesis may be determined during perinatal life. It is tempting to speculate that a reduced level of neurogenesis, secondary to early stress exposure, may contribute to maladaptation of the HPA axis and possibly to the increased vulnerability of women to stress-related disorders.

## Introduction

A multitude of studies have implicated alterations in the hypothalamo-pituitary-adrenal (HPA) axis in the vulnerability to develop stress-related disorders like major depression [1]–[7]. One of the brain areas sensitive to stress and stress hormones is the hippocampus, a region involved in learning and memory, behavioral adaptation and HPA-axis regulation [8]–[10] and richly endowed with glucocorticoid (GR) and mineralocorticoid receptors (MR), [11].

Chronic exposure to stress can affect both hippocampal function and structure. Consistent reductions in hippocampal volume have e.g. been reported in major depression, as a predictive factor rather than a consequence of the disorder [12]. Although the functional implications and the biological substrates that underlie hippocampal volume reductions are ill understood, animal studies have shown that chronic stress can induce cellular and dendritic atrophy, alter glia cell numbers and reduce adult neurogenesis [13]–[20]. Adult hippocampal neurogenesis represents a form of structural plasticity that has been implicated a.o., in hippocampal function [21]–[25] and the efficacy of antidepressants [17], [26]–[30]. Stress and glucocorticoids potently inhibit neurogenesis in adult animals [18]–[20], [31]–[33].

Major depression is more prevalent in women than in men [34]–[38]. The neurobiological mechanisms that could account for this difference are not well identified, but recent data show that hippocampal volume reductions in depressed women occur only when depression is preceded by an early life stressor [39]. This underlines the potential importance of early life stress, at least in women, for vulnerability to develop depression.

In rodents, exposure of the pregnant dam to stress affects critical periods of fetal brain development that can persistently alter structural, emotional and neuroendocrine parameters in the offspring [40]–[49]. This results e.g. in altered anxiety-like behavior, increased hypothalamic-pituitary-adrenal (HPA) axis reactivity and memory deficits in adult life [50], [51]. Prenatal restraint stress was further shown to reduce dentate granular cell number, but only in female offspring [52], while also other sex-related behavioral and structural differences have been reported [48].

Postnatally, several manipulations have been shown to shape various stress and HPA-axis parameters [53]–[56]. In rats, from postnatal day (PND)3–4 to PND14 of life, basal ACTH and corticosterone levels are kept low and the response to most stressors is suppressed [57], [58]. Escape from this suppression is seen after maternal deprivation (MD), an early life stressor during which pups are separated from their mother. Single, 24 h maternal deprivation in rats resulted in increased basal corticosterone levels in young [59], but not older rats [53], [60], [61]. Also, stress-induced ACTH [59], [61] and corticosterone responses were increased in 24 h MD rats [53], [60] while similar patterns are found in repeated separation paradigms [56], [62]–[65]. Most of these endocrinological analyses were performed on male offspring, but female specific effects occur as well [66]–[69].

In addition, postnatal stress affects hippocampal structure [70]–[73]. Maternal deprivation or low levels of maternal care reduces hippocampal neurogenesis in some [73], [74], but not all [75] studies. As the dentate gyrus of the hippocampus is largely formed postnatally [76], [77], effects on neurogenesis and structural plasticity are potentially more pronounced and longer lasting when stress is applied early in life [45], [78].

Since early life stressors are important, at least in women, for the development of depression, we here tested the hypothesis that 24 h of MD at PND3 differentially affects hippocampal structural plasticity in female versus male offspring. Postnatal day 3 was chosen as a timepoint for maternal deprivation, since this day represents the start of the development of the tertiary matrix of granular cells during dentate gyrus formation [76], [77], [79]. This tertiary matrix produces the inner shell of the granular cell layer, i.e. the future site of adult neurogenesis. We expect this timepoint will have considerable impact on dentate gyrus neurogenesis, structure and possibly also function later in life. We stereologically analyzed newborn cell proliferation and survival as well as neurogenesis at PND21, an age at which we expect both short-lasting as well as chronic effects of MD to be detectable. Given recent studies showing stress effects on glia cell numbers [13], we also analyzed the total number of GFAP-positive astrocytes and the extent of astrogliogenesis.

## Materials and Methods

### Animals and breeding procedure

All experimental procedures were approved by the local animal committee of the University of Amsterdam. To standardize the perinatal environment, rats were bred inhouse. Thirteen male and 26 female Wistar rats (3 months old) were purchased from Harlan (Zeist, the Netherlands) and habituated to the animal facilities for 10 days. Animals were housed in pairs with food and water available ad libitum. During the entire experiment, rats were put on a 12 h light/dark cycle (lights on at 8.00 a.m.), at 20°C with 40–60% humidity. After habituating, breeding started. Two females were housed together with 1 male for one week after which the male was separated from the females. Females were then housed together for another week after which they were separated. Females were daily observed for pups and when a litter was found before 9.00 a.m., the previous day was designated as the day of birth or postnatal day (PND) 0. Litters were left undisturbed until PND3 and were then randomly assigned to one of the four groups, taking into account that litters from one male were not included in the same experimental group.

### Groups and experimental design

Maternal deprivation for 24 h does not only result in the absence of maternal care, but also in lack of nutrition, which is considered a physiological stressor. To control for this, additional experimental groups were included in which deprived pups were injected with glucose (maternally deprived+glucose; MDG). This has been previously described to delay the onset of the HPA-axis activation in mice during an MD procedure of 8 h [80]. To control for the stress of glucose injections, two other groups were sham-injected (control sham; CONS and maternally deprived sham; MDS) in addition to a non-injected control group (control undisturbed; CONU).

Pups from all four experimental groups (CONU, CONS, MDS, MDG) were sacrificed on PND21 and brains were used to study hippocampal neurogenesis. In order to asses effects of MD on newborn cell survival, bromodeoxyuridine (BrdU; 75 mg/kg; subcutaneous) was injected on PND3 (see below).

### Maternal deprivation

All four groups were left undisturbed until PND3. On the morning of PND3, one hour after the onset of the light-phase, the dam was taken from the nest and placed in a clean cage. To minimize stress for the dam, her cage was returned to the same room. To avoid disturbance by the vocalization of the pups [81], the home-cage with the litter was moved to another room and placed on a heating pad and the litter was kept on a temperature of 32°C for the rest of the 24 h. During deprivation, glucose (200 mg/kg bodyweight, in a volume of 5 µl per gram bodyweight) was administered to MDG pups three times, starting at 2 hours after the onset of deprivation (11.00 am), and additionally at 17.00 and 22.00h to compensate for the lack of nutrition during the full 24 h. The timepoint of first injection was used to additionally inject the birth date marker BrdU in a similar injection volume for all four groups. Control litters were left either undisturbed (with the exception of a BrdU injection), (CONU), or received sham injections at all timepoints (CONS). Sham-injections to CONS animals were performed in a minimum amount of time, during which the mother was briefly placed in another cage. All injections were given subcutaneously with a 50 microliter Hamilton syringe (33 Gauge, Hamilton, Switzerland) in the skin of the neck.

The following day at 9.00 am, MDS and MDG animals were weighed, culled to 4 males and 4 females per litter and placed back with their mother. The CONS litters were also weighed and culled to 8 animals, but the CONU group was left undisturbed.

### Acute effects of MD

To determine the acute effects of MD on corticosterone levels, surplus animals from culling and a subset of four litters were sacrificed by rapid decapitation at 9.00 am on PND4. Blood samples were collected in EDTA-containing tubes, placed on ice and subsequently centrifuged at 5000 rpm for 20 minutes after which the supernatant was stored at −20°C. Plasma corticosterone levels were measured by means of a radioimmunoassay (MP Biomedicals., Amsterdam, the Netherlands). In addition to blood samples, PND4 brains were taken out rapidly and fixed by immersion fixation in 4% paraformaldehyde in phosphate buffer (PB 0,1 M; pH 7.4) for 48 hours. Afterwards, brains were stored on PB with azide until further processing.

### Maternal care observations

Previously, maternal care has been shown to be increased towards the male pups [82] and to be affected by early handling and deprivation [83], [84]. As neurogenesis could be possibly affected by changes in maternal care [74], we therefore assessed whether changes in the amount of maternal care directed to either male or female pups after MD had occurred. To differentiate between male and female pups, all litters were marked on a daily basis. Since marking itself represents an additional handling procedure, offspring of these experiments was not used for further analysis. Two of the previously described experimental groups were included in this experiment: the 24 h deprived group receiving sham-injections (DS, n = 6 litters) and the control group receiving sham injections (CS, n = 5 litters). All experimental procedures were the same as in the original experiment, except that all pups were labeled with a non-toxic surgical marker (Codman) daily on either the upper part of the body or the lower part to separate the sexes. To exclude any effects of marking either the lower or the upper part of the body, this was alternated for males and females between litters.

Maternal care observations were performed as described earlier [85]. Maternal behavior of each dam was observed for five 60-minutes observation periods per day, from PND1 until PND7. Observation periods took place at 7.00 am, 10.00 am, 1.00 pm, 5.00 pm, and 8.00 pm, resulting in two dark-phase observations and three light-phase observations. On each day, marking of the pups took place within 10 minutes per litter, immediately following the first (7.00 am) observation period. During each 60 minute observation period, behavior was scored every 3 minutes, resulting in 20 observations per period and 700 observations in total (PND1–PND7) for the CS-group and 600 observations in total for the DS-group, since PND3–4 was lacking. Licking and grooming (with or without nursing) was scored for male and female pups. The scoring of other behaviors, however, was done for the litter as a whole and included: a) arch back nursing (defined as the dam displaying an obvious arc in her back while nursing), b) blanket nursing (dam lies flat on her pups while nursing), c) passive nursing (dam lies on her side), d) self grooming of the dam and e) time away from the litter.

On PND3, six litters were deprived from their mother for 24 h, according to the procedures described above. The control (CS) litters remained with their mother. Both CS and DS groups received sham injections at 11.00 am, 5.00 pm and 10.00 pm to replicate the procedures of the groups studied for neurogenesis as closely as possible. This resulted for the CS group in two observation periods that preceded injection times (11.00 am and 10.00 pm) and in one observation period that followed injection time (5.00 pm). On the following morning, i.e. on PND4, DS litter were placed back with their mothers at 9.00 am, which was followed by the 10.00 am observation period.

### Perfusion and tissue processing

On PND21 rats were anaesthesized in the morning by an injection of pentobarbital sodium salt (Nembutal, 1 mg/kg bodyweight; A.U.V. Cuijk, The Netherlands) and perfused transcardially with saline followed by 4% paraformaldehyde in phosphate buffer (PB; 0.1 M; pH 7.4). To prevent pressure artefacts, brains were additionally postfixed overnight within the skull at 4°C, washed and cryoprotected in 20% sucrose in PB. Frozen sections (30 µm thick) were cut using a sliding microtome and collected in PB with azide.

### Immunohistochemistry

Different stages of neurogenesis were studied as described previously [17]. Immunohistochemistry for BrdU (monoclonal murine anti-BrdU, Roche Diagnostics, Netherlands, 1∶2000) was used to assess newborn cell survival, Ki-67 (polyclonal rabbit α-Ki-67, Novocastra, New Castle, UK, 1∶2000) to assess proliferation, and doublecortin (DCX; polyclonal goat α-DCX, Santa Cruz, 1∶800) to assess the number of immature neurons. To analyze astrocyte numbers and astrogliogenesis in the dentate gyrus, immunohistochemistry for GFAP (polyclonal goat anti-GFAP, DAKO 1∶10000) was done as well. The primary antibody was amplified by biotinylated rabbit anti goat (Vector); avidin-biotin enzyme complex (ABC kit; Elite Vectastain, Brunschwig Chemie, Amsterdam, 1∶1000) and developed with diaminobenzidine (DAB; 20 mg/100 ml tris buffer; TB, 0.01% H_2_O_2_). For BrdU/GFAP double-labeling, protocols were combined. After first developing BrdU immunoreactive signal with nickel ammonium sulphate (0.02%) added to the diaminobenzidine (DAB; 20 mg/100 ml TB, 0.01% H_2_O_2_), sections were subsequently incubated in GFAP primary antibody (polyclonal goat anti-GFAP, DAKO 1∶10000) overnight. The next day, GFAP antibody was amplified according to standard protocols using secondary biotinylated rabbit anti goat (Vector) and avidin-biotin complex (ABC kit; Elite Vectastain, Brunschwig Chemie, Amsterdam, 1∶1000). For GFAP, chromogen development was by diaminobenzidine (DAB; 20 mg/100 ml TB, 0.01% H_2_O_2_) alone.

### Stereology

GFAP+, DCX+ and BrdU+ cells were quantified stereologically by systematic random sampling in every 10^th^ section using the StereoInvestigator system (Microbrightfield, Germany) in a total of 9 sections per animal. Because of the occurrence of cell clusters when using Ki-67, all individual positive cells were counted by means of a modified stereological procedure, manually in every 10^th^ hippocampal section (Zeiss microscope 200× magnification) and multiplied by 10 to estimate the total number of Ki-67+ cells per hippocampus.

To determine the percentage of BrdU-labeled astrocytes as part of the whole BrdU cell population, random sampling was done in six hippocampal sections per animal. In each section, two randomly selected parts of the dentate gyrus were sampled throughout the granular cell layer, the subgranular zone and the hilus, and the ratio of BrdU/GFAP double-positive over the number of BrdU-single positive cells was expressed as a percentage. Dentate gyrus granular cell layer volume measurements were performed by using the Cavalieri estimator in every 10^th^ section in a total of 9 sections per animal.

### Statistics

Data are presented as mean+SEM. All statistics were performed by SPSS16 for Mac. Immunohistochemical data were initially compared using a two-factor ANOVA to study the main effect of sex and treatment. In case of a significant interaction between sex and treatment, a one-way ANOVA over the four treatment groups was performed, separately for male and female data. If the one-way ANOVA revealed significance, a pair wise comparison was performed with a post-hoc LSD test. Maternal care data was analyzed with a one-way ANOVA per day in case of individual licking and grooming; for arch-back nursing an ANOVA for repeated measures was used.

## Results

### Acute effects of 24 h MD on PND4

#### Body weights and corticosterone levels

Animals were not labeled to avoid any disturbance in the control groups. Therefore, individual bodyweight data are unavailable. However, from P3 to P4, both MD groups experienced on average a 4% weight-loss, as opposed to an average weight gain of 7% in the CONS group. The undisturbed groups were not weighed on PND4.

**Figure 1:**
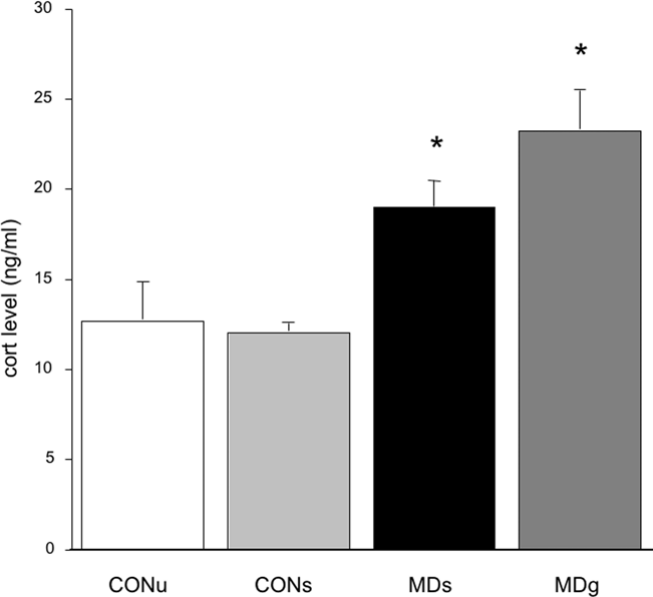
Basal corticosterone levels on PND4. A significant increase is found in corticosterone levels after maternal deprivation (MDs, sham injected) compared to controls (both undisturbed CONU and sham-injected CONS). Additional glucose treatment (MDG) failed to normalize this (F_(3,55)_ = 10.30; p<0.0001; post-hoc: CONU = CONS<MDS = MDG at least p<0.05).

Maternally deprived pups in both MD-groups had significantly higher morning corticosterone levels when compared to controls (F_(3,55)_ = 10.30; p<0.0001; post-hoc: CONU = CONS<MDS = MDG at least p<0.05; see figure 1). As there were no differences between the sexes in corticosterone level at this age, data were pooled for males and females.

Overall, MD had appreciable immediate effects on body weight and corticosterone level. Importantly though, the sham-injected control pups responded in a comparable manner as the pups that were left undisturbed. Moreover, repeated administration of glucose during the 24 MD period did not ‘rescue’ the phenotype.

#### Dentate gyrus cell proliferation

**Figure 2:**
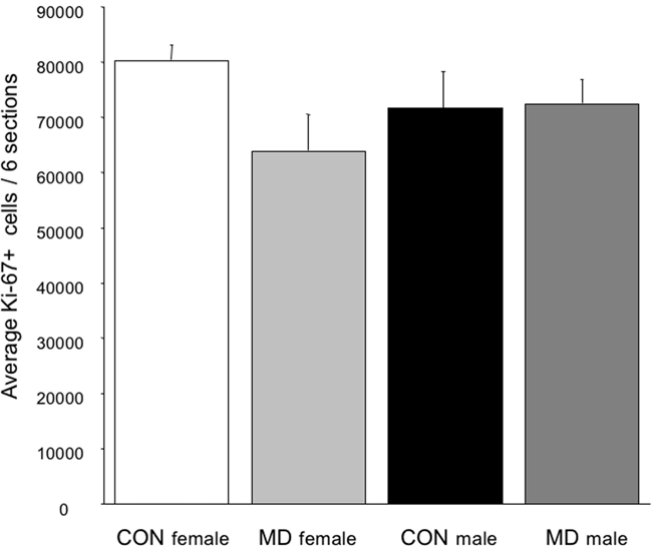
Dentate gyrus cell proliferation (Ki-67) on PND4 in CONS and MDS animals. There was no significant effect of sex (F_(2,21)_ = 0.04; p = 0.95) or treatment (F_(3,21)_ = 1.13; p = 0.30) and no interaction between the two (F_(3,21)_ = 2.45; p = 0,14).

In a subset of animals, dentate gyrus cell proliferation was measured immediately after 24 h MD, i.e. on PND4. For this experiment CONS males and females and MDS males and females were used. Despite the effect of MD on body weight and circulating corticosteroid levels, no acute effects of treatment were observed on dentate gyrus cell proliferation, as measured by total Ki-67+ cell population (F_(3,21)_ = 1.13; p = 0.30), see figure 2. A two-factor ANOVA revealed no effect of sex on proliferation (F_(2,21)_ = 0.04; p = 0.95) or an interaction between sex and treatment (F_(3,21)_ = 2.45; p = 0,14).

### Effects of early MD on structural parameters at PND21

#### Body weights

There was a significant difference in body weight between the 4 experimental groups at PND21 (F_(3.91)_ = 50; p<0.0001). In the control undisturbed group (CONU), body weights were the lowest (mean+SE: 45+0.6). CONS animals were heavier (57+0.9), compared to all the other groups, and MDS and MDG animals had lower body weights than the CONS group but did not differ from each other (52+0.7 and 49+1.2 respectively). Post-hoc analysis revealed that CONS animals were significantly heavier than the MDS (p<0.05), as were the MDG compared to CONU rats. The two MD groups did not differ from each other.

#### Granular cell layer volume

**Figure 3:**
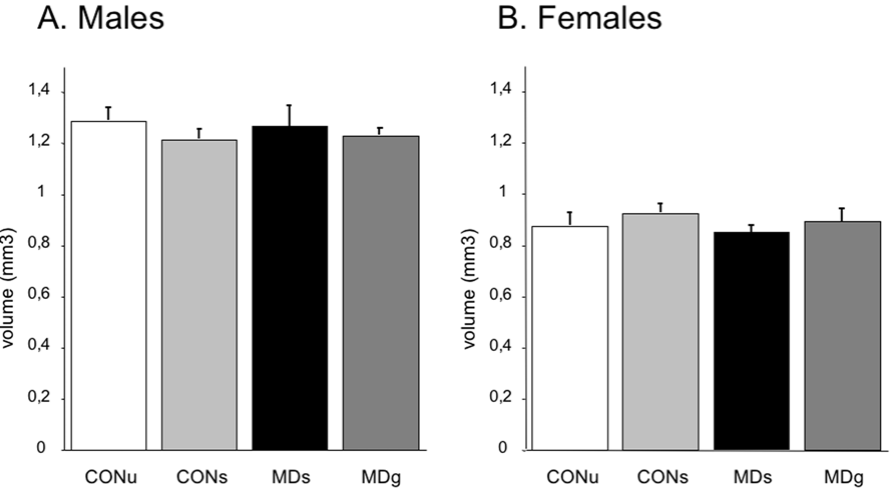
Granular cell layer (GCL) volume on PND21. There was no effect of any of the treatments on granular cell layer volume. However, significantly lower GCL volumes were found in females (F_(1.51)_ = 122.4; p<0.0001).

The volume of the granular cell layer was not affected by MD with or without glucose administration, neither in males (F_(3.28)_ = 0.41; p = 0.75) nor in females (F_(3.28)_ = 0.54; p = 0.65) as shown in figure 3. A two-factor ANOVA revealed a significant effect of sex on granular cell layer volume (F_(1.51)_ = 122.4; p<0.0001), female offspring showed a lower average granular cell layer volume than males. No significant interaction of treatment and sex was found (F_(3.51)_ = 0.95; p = 0.42).

#### Proliferation, newborn cell survival and neurogenesis

**Figure 4:**
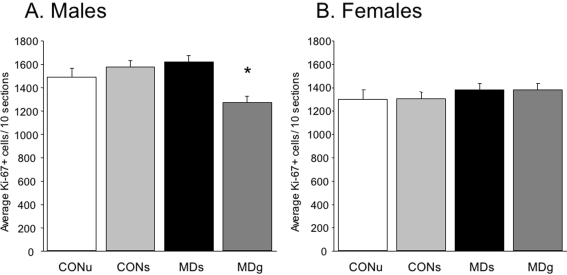
Dentate gyrus cell proliferation (Ki-67) on PND21. A. In males, MD affected dentate cell proliferation rate at PND21 (F_(3,28)_ = 3,2, p = 0.043). Post-hoc analysis revealed a decrease in MD-glucose injected animals (p<0.05). B. In females, there was no effect of treatment (F_(3,28)_ = 0.41 p = 0.75), although a significant lower number of Ki-67 positive cells was found in females when compared to males (F_(1,56)_ = 8.56; p = 0.005).

A two-factor ANOVA revealed no main effect of treatment on proliferation (F_(3,56)_ = 0.72; p = 0.55) but a significant effect of sex (F_(1,56)_ = 8.56; p = 0.005) and a significant interaction (sex×treatment) was found, indicating that the effects of MD are different in males than in females (F_(3,56)_ = 2.88; p = 0.045). Females have overall lower numbers of Ki-67 positive cells. A one-way ANOVA per sex revealed that MD affected dentate cell proliferation in males (F_(3,28)_ = 3.2; p = 0.043) but not in females (F_(3,28)_ = 0.41; p = 0.75, see figure 4). Only the MDG males had significantly decreased numbers of Ki-67 positive cells (post-hoc comparison, p<0.05).

**Figure 5:**
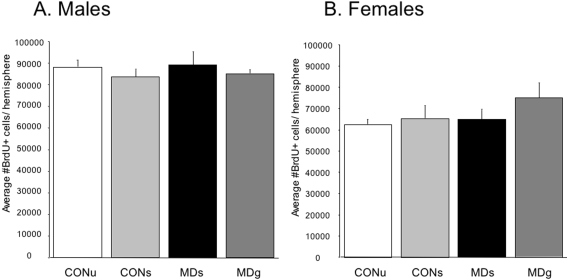
Number of newborn surviving cells (BrdU+) in the dentate gyrus on PND21. There was a significant effect of sex, but not of treatment on BrdU positive cell numbers. A. Maternal deprivation did not alter BrdU+ cell numbers in males (F_(3,28)_ = 0.40, p = 0.75), or (B) females (F_(3,28)_ = 1.1, p = 0.37). Irrespective of MD, females had an overall lower number of BrdU-positive cells (F_(1,51)_ = 29.8; p<0.0001).

On PND21, survival of newborn cells in the dentate gyrus was not affected by MD in males (F_(3,28)_ = 0.40; p = 0.75), or females (F_(3,28)_ = 1.1; p = 0.37), see figure 5. There was a significant effect of sex on cell survival. Overall, female offspring had a lower number of 17 day old BrdU+ cells in the dentate gyrus than males (F_(1,51)_ = 29.8; p<0.0001). No significant interaction of treatment and sex was found (F_(3.51)_ = 0.93; p = 0.43). During counting the location of the cells within the dentate gyrus, i.e. subgranular zone, granular cell layer or hilus, was taken into account, but this did not yield any subregion-specific effects. When corrected for granular cell layer volume, all of the above effects persisted (data not shown).

**Figure 6:**
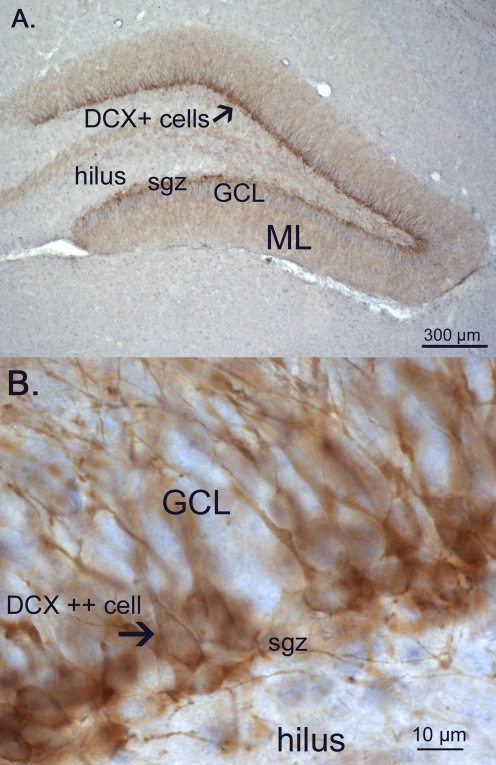
Doublecortin (DCX) -positive neurons. A. Photo of the dentate gyrus of a 21 day old CONS male showing extensive immunostaining of DCX in the subgranular zone (sgz) and the first third of the granular cell layer (GCL) with dendrites extending through the granular cell layer (GCL) into the molecular layer (ML). B. High power photomicrograph showing details of the DCX+ cell bodies located in the SGZ and GCL, with extending dendrites in the GCL.

**Figure 7:**
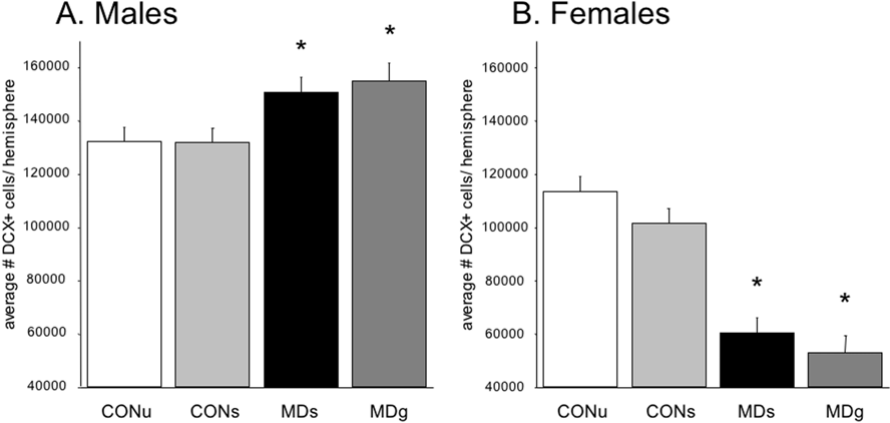
Doublecortin (DCX) -positive neuron numbers on PND21. A significant treatment×sex interaction revealed a differential effect of MD on males versus females (F_(3,48)_ = 8.04; p<0.0001). A. An increase in DCX+ cell number was found in deprived males (F_(3,28)_ = 4.3, p = 0.018; post-hoc: CONU = CONS<MDS = MDG, at least p<0.05) and a decrease in deprived females (F_(3,28)_ = 4.65, p = 0.013; post-hoc: CONU = CONS>MDS = MDG, at least p<0.05) when compared to controls. A significant effect of sex indicates a general lower amount of DCX+ cells in females (F_(1,48)_ = 65.80; p<0.0001).

The most prominent effects of MD were observed at PND21 in neurogenesis, as measured by the young neuronal marker doublecortin (DCX; figure 6), A two-factor ANOVA revealed no main effect of treatment (F_(7,48)_ = 1.50; p = 0.23), but an effect of sex, indicating that females have in general less DCX-positive cells (F_(1,48)_ = 65.80; p<0.0001). Interestingly, a significant interaction between sex and treatment was found, indicating that the effects of MD on neurogenesis were different for males than females, (treatment×sex; F_(3,48)_ = 8.04; p<0.0001). A one-way ANOVA in males, revealed a significant increase in the total number of DCX+ cells due to maternal deprivation (F_(3,28)_ = 4.3; p = 0.018; post-hoc LSD: CONU = CONS<MDS = MDG, at least p<0.05, see figure 7A). In females, MD was found to lead to *lower* total DCX+ cell numbers, (F_(3,28)_ = 4.65; p = 0.013; post-hoc LSD: CONU = CONS>MDS = MDG, at least p<0.05 see figure 7B). When corrected for granular cell layer volume, these effects persisted (data not shown).

#### Astrocyte numbers and gliogenesis

**Figure 8:**
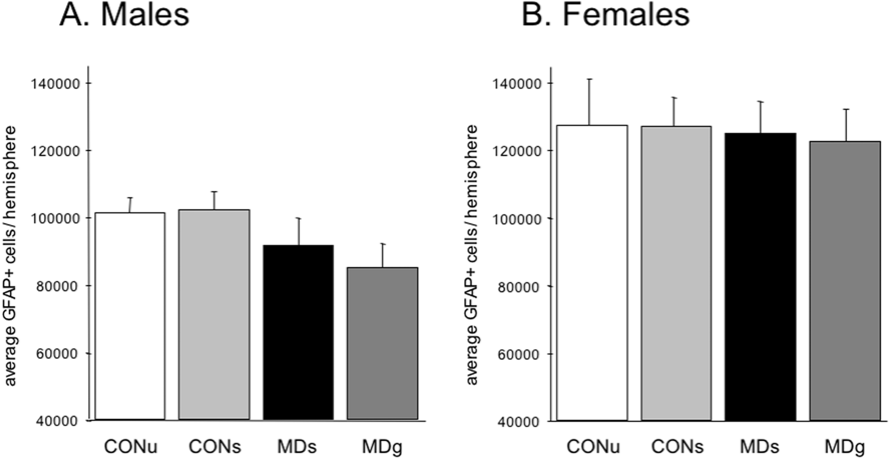
Astrocyte numbers. GFAP-positive astrocyte numbers determined stereologically in the entire hippocampal dentate gyrus on PND21. A. Maternal deprivation did not affect GFAP+ cell number in males (F_(3,28)_ = 1.3, p = 0.28), B. nor females (F_(3,28)_ = 0.05, p = 0.99). However, a significant effect of sex on GFAP+ cell number was found (F_(3,56)_ = 25.01; p<0.0001).

The total number of GFAP+ astrocytes was measured in a stereologically sampled series using the StereoInvestigator throughout the entire dentate gyrus; in the hilus, granule cell layer as well as molecular layer. No effects of early MD were found at PND21 on the total number of GFAP+ cells in males (F_(3,28)_ = 1.3; p = 0.28), nor in females (F_(3,28)_ = 0.05; p = 0.99, see figure 8). A two-factor ANOVA revealed a significant effect of sex (F_(3,56)_ = 25.01; p<0.0001) but no interaction of treatment and sex (F_(3,56)_ = 0.23; p = 0.88). Females show a significant *higher* number of GFAP-positive cells compared to males.

**Figure 9:**
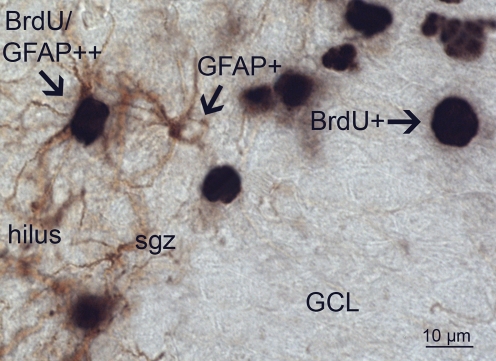
GFAP/BrdU double labeling. Immunohistochemical double labeling for GFAP and BrdU shows single GFAP+ astrocytes in the hilus with their processes occasionally extending into the sgz. GFAP+ cells reveal brown DAB-staining in their processes and cytoplasm whereas the nucleus is devoid of staining. In the GCL, BrdU+ single cells are stained black by DAB-nickel as indicated (BrdU+) in the granular cell layer (GCL). Shown on the left is a BrdU/GFAP double labeled cell (arrow).

**Figure 10:**
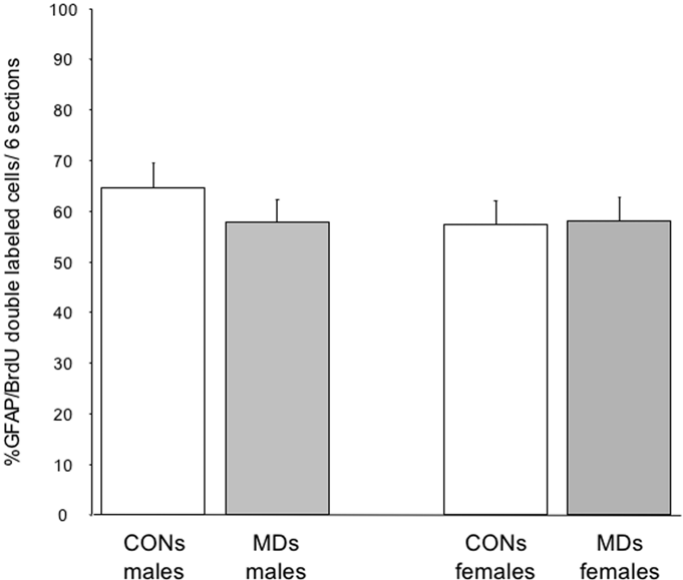
Percentage of GFAP/BrdU double-labeled cells in the dentate gyrus. There was no effect of MD on the percentage of double labeled cells in both males and females (F_(3,24)_ = 0.40; p = 0.54). No effect of sex was found (F_(1,24)_ = 0.56; p = 0.46).

Quantification of gliogenesis was done by analyzing BrdU and BrdU/GFAP double stained cells (for an example, see figure 9). Random sampling of a minimum of 200 BrdU positive cells per animal in six hippocampal sections was done, and the percentage double labeling with GFAP is shown in figure 10. In this experiment, only CONS and MDS males (n = 6) and females (n = 6) were analyzed. There was no effect of MD on the percentage of double labeled cells (F_(3,24)_ = 0.40; p = 0.54) no effect of sex (F_(1,24)_ = 0.56; p = 0.46) and no interaction between sex and treatment was found (F_(3,24)_ = 0.6; p = 0.45).

### Maternal care

**Figure 11:**
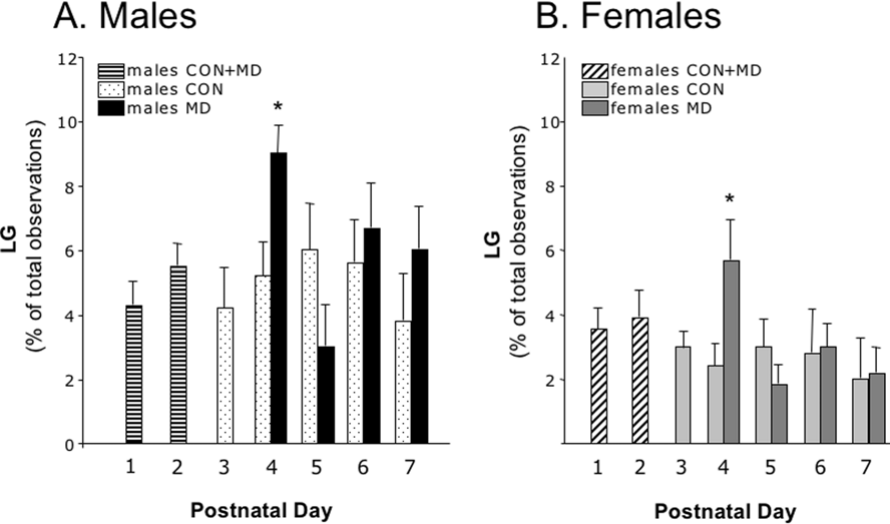
Licking and grooming on PND1–7 in control and MD litters. A significant increase in LG was found on PND4 both in males and females compared to their same-sex controls. (PND4 LG-scores: males F_(3,21)_ = 7.31; p = 0.002; post-hoc: MD males>CON males = MD females>CON females, p<0.05).

As neurogenesis could possibly be affected by maternal care [74], we therefore assessed whether possible changes after MD in the amount of maternal care directed to either male or female pups could explain the outcome of this study. As expected [84], maternal care was affected 24 h after MD (figure 11) and dams provided more care towards the pups when returned to the nest, as illustrated by the increased percentage of time spent on licking and grooming (LG). Both MD-males and MD-females received significantly more care (LG) on PND4 compared to the respective control animals. In total, males received more individual care than females (males: 5.6%±0,4; females 3.2%±0.3; T-test, p = 0.008). This effect was also seen on PND4, because the percentage of LG towards male and female pups increased similarly, which brought the MD females to the level of non-deprived males (PND4 LG-scores: males F_(3,21)_ = 7.31; p = 0.002; post-hoc: MD males>CON males = MD females>CON females, p<0.05, see figure 11).

**Figure 12:**
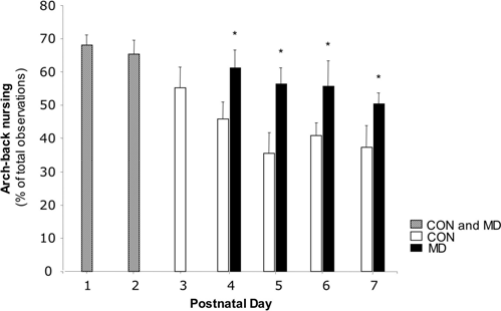
Arch back nursing. Arch back nursing towards the entire litter in control and MD litters on PND1–7. There is a significant increase after PND3 (F = 7.89, p = 0.02).

When comparing the amount of active nursing by the dam towards the whole litter, as measured by arch-back nursing (ABN), MD resulted in a significant increase of ABN after PND3 (repeated measures ANOVA; F = 7.89, p = 0.02, see figure 12).

## Discussion

We show that 24 h of maternal deprivation at PND3 alters hippocampal structural plasticity in a sex-dependent manner. Although newborn cell survival and proliferation rate were not altered by MD, neurogenesis in the dentate gyrus was increased in male, but decreased in female offspring. Since no such differential changes were found in granular cell layer volume, astrocyte number or astrogliogenesis, this indicates that instead of altering granule cell numbers, MD-induced stress elicits specific changes in subsets of the differentiating cell population and e.g. impacts only the immature, DCX positive cells.

### Neurogenesis specific effects of MD

Given the extensive neurogenesis during gestation and the early postnatal period [86], [87], it is not surprising that early life stress affects structural brain development. Indeed, long-lasting reductions in neurogenesis and hippocampal functions after both pre- as well as postnatal stressors have been reported in most [41], [45], [46], [73], [88], but not all [89] studies. Stress-induced increases in maternal and offspring plasma corticosterone levels during a sensitive time window of brain development appear to be critical in mediating these long-lasting effects [51], [90], [91]. The timepoint of early life stress as was used in the present model coincides with the formation of the inner shell of the dentate granular cell layer i.e. the future site of adult neurogenesis [76], [77], [79]. The present results show that for the rat dentate gyrus, the sensitive time window during which brain development can be affected, appears to extend at least into the early postnatal period.

Despite the significantly different corticosterone levels at the end of the 24 h MD period, no changes were found in the numbers of Ki-67+ proliferating cells at PND4. In adult rats, stress frequently reduces proliferation [19], [92]–[94] but clear exceptions have also been reported [20], [95]–[97] that may depend on the experimental design and type of stress [33]. If any reduction was induced during the 24 hours of MD, recovery was fast and compensated for, or normalized, rapidly. However, in MD males that were additionally given a glucose injection, an unexpected reduction in proliferation was found. It can be speculated that a protective response of the pup during MD is disturbed by metabolic activation and a subsequent insulin response. Although there is considerable evidence for a strong and complex interplay between the metabolic system and the (development of) the HPA-axis [80], [98], the exact mechanisms can not be derived from the current data-set and await future studies. Given the very large numbers of newly generated cells at these young ages, also stochastic differences within the population could have occurred that may have been missed with Ki-67 immunohistochemistry as this antibody labels all cells engaged in cell cycle.

Even though the sex differences in hippocampal neurogenesis could be attributable to differential precursor kinetics, this has not led to reductions in granular cell layer volume between MD and control females. Also, no changes were found in the number of BrdU+ surviving cells. Various stress protocols have been shown to reduce single or multiple phases of the neurogenic process, but only in a few instances, and when stress or corticosterone exposure was chronic, did this actually lead to reductions in DG granule cell number [99]. In addition to the MD-induced differences between male and females, also differences between non-deprived control male and females were present. So far, not much is known about sex-differences per se in DG neurogenesis measured around the age of weaning and thus before the onset of the oestrus cycle. However, our results show that control males generally have a higher proliferation rate, an increased survival of newborn cells, more young neurons (DCX) but less astrocytes then control females. In addition, a larger granular cell layer volume was found in males.

Furthermore, an extensive amount of literature has shown differential regulation by gonadal hormones [92], [100]–[107], resulting in established sex differences in proliferation and survival. Also, a differential response to chronic stress in males and females was found [92], [106]. Typically, under basal conditions, both estradiol and testosterone enhance neurogenesis [105], [108], [109]. In adult female rats, an increased proliferation rate occurs during pro-oestrus [105] due to higher estradiol levels. Males were found to have more doublecortin positive cells [47]. During development, sex steroids are also able to modulate astrocytes [110] and a higher number of hippocampal astrocytes in females was found in some [111], [112], but not all [113] studies. In summary, our data indicate that sex-differences in dentate gyrus structure and neurogenesis are already present before sexual maturity is reached.

In contrast to proliferation and newborn cell survival that remained unchanged, neuronal differentiation was differentially altered between males and females, as based on DCX immunohistochemistry. One possibility to explain these differential changes in DCX could be a change in cell-fate determination. In deprived males, more newborn cells may have differentiated into a neuronal phenotype, as compared to deprived females, e.g. at the cost of gliogenesis. The latter option is unlikely, since no compensatory changes in either total astrocyte cell numbers or astrogliogenesis were found. An additional option is a shift towards oligodendrogliosis, but although these numbers were not determined, the proportion of newborn cells that differentiate into oligodendrocytes is generally very small [114].

Another possibility is that the differential changes in DCX+ cell numbers are related to the time window of DCX expression. DCX is a microtubule associated protein (MAP) expressed by migratory and immature neurons from PND 4 till 14 and accurately reflects neurogenesis in the adult hippocampus [115], [116]. In theory, MD may shorten the time window during which DCX is expressed, which would imply that when shut-off prematurely, it could lead to an early arrest in DG granule cell development and lower DCX+ cell numbers in females, which would result in less complex granular cells. The finding that the number of BrdU-+ cells did not differ between male and female groups, indicates that the DCX changes are at least not accompanied by a changed survival rate or different developmental kinetics of the newborn cell population.

Instead of altering neuron number, MD may specifically impact the population of immature DCX-IR cells. Preliminary data indicate that in adult females, the complexity of the individual granule cell is indeed diminished (unpublished results). Whether this has consequences for functional properties of the hippocampal circuit awaits future research. Following the same line of reasoning, males could benefit from maternal deprivation on PND3, as this resulted in an increase in neurogenesis. It is known that different early life experiences can cause differential responses in stress reactivity in adulthood. Whether consequences are detrimental or more beneficial, depends to a large extend on the context the adult subject encounters [117]. It not only remains to be shown in future studies whether the present differences in neurogenesis are transient, but also whether MD-induced changes in neurogenesis correlate with later structural and functional parameters in a more positive way in adult male offspring.

### Sex differences in the effects of MD on neurogenesis

Recent studies have revealed sex-dependent alterations in DCX expression [47] after prenatal restraint stress exposure. Similar to our study, higher DCX expression was found in males. However, in this study, no effects of prenatal stress on total DCX cell number were found in females. The interaction of stress hormones with gonadal steroids during gestation may explain these results [48]. In the present study, however, both male and female offspring experienced an increase in corticosterone not until PND4 and gonadal interactions during pregnancy are therefore unlikely. However, also in adulthood, gonadal steroids affect hippocampal plasticity to a great extent. For example, stress experienced in adulthood decreases proliferation and neurogenesis in males, but not females, and estrogen is thought to protect against stress-induced reductions in dentate gyrus proliferation [92], [105], [106]. Estrogens can exert non-genomic effects directly and indirectly on newly generated cells in neonatal and adult rat dentate gyrus while specific estrogen receptors are found on DCX+ cells, which is interesting in this respect [118]. Testosterone on the other hand, promotes neurogenesis and survival but not differentiation of the newborn cells [101], [119]. Although gonadal steroids may hence contribute to the development of sex differences in neurogenesis per se, it awaits to be determined whether they are also implicated in the differential effects of MD in PND21 animals.

Body weight and basal corticosterone levels were affected by MD but, interestingly, these measures were not altered by glucose supplementation, nor by the multiple injections (of glucose or vehicle) associated with the treatment. Importantly, though, corticosteroid levels were not different between males and females. This does not exclude, however, that other factors determining corticosteroid functionality change in a sex-dependent manner. Thus, sex-specific effects were reported for MR and GR expression after 24 h MD on PND3 [120]. In males, the same MD design reduced GR and MR binding whereas in females, GR was upregulated and MR was unaffected [120]. Selective upregulation of hippocampal GR after MD in females could sensitize young neurons to the actions of circulating corticosterone, and may e.g. result in a premature shut-down of DCX expression. Also, MD may differentially affect early HPA axis (re)activity. The DG may be particularly vulnerable then as it undergoes rapid postnatal development during the first two weeks of life [76], [77], [87]. Whether or not the newborn cells are actually sensitive to glucocorticoid action depends on the GR and MR expression of the individual newborn cell. As documented elsewhere [121], newly formed, proliferating cells only express low and variable levels of GRs, which may explain the lack of effect of CORT on proliferation. Considerable expression levels of both GR and MR on the individual newborn cells are only reached in young neurons [121], consistent with the present DCX findings.

Finally, since maternal behavior shapes hippocampal properties later in life [83], [117], we investigated whether sex-specific differences in maternal care had been instrumental in the changed pattern of neurogenesis. Maternal behavior regulates maturation of offspring HPA activity [57], [83] while stress affects maternal-offspring interactions [84]. Consistent with earlier findings [82], we also found a higher level of licking and grooming towards male than female pups. Individual maternal care after MD was increased on PND4, but this increase was comparable for male and female pups and returned to control levels from PND5 onwards. Maternal care through arch back nursing was indeed enhanced by MD but this was towards the entire litter. Therefore, it is unlikely that the male-female differences in neurogenesis can be explained by sex-specific changes in LG or other maternal behavioral components induced by MD.

Taken together, the present data support the concept that the setpoint for neurogenesis may be determined during perinatal life and illustrate the critical influence of early environment on establishing sex differences in neural plasticity. They expand our understanding of the mechanisms underlying sex differences and highlight the critical role early stress can play in determining the structural make up of the hippocampus in adulthood. Given their specific properties [122], newborn cells can make disproportionately large contributions to overall DG composition, average age and output of the DG cells, which will have considerable consequences for hippocampal function [21], [123]. It is tempting to speculate that a reduced level of neurogenesis, secondary to e.g. early stress exposure, may contribute to maladaptation of hippocampal function and possibly to increased vulnerability of women to stress-related disorders.
